# Glanders outbreak at Tehran Zoo, Iran

**Published:** 2012-03

**Authors:** P Khaki, N Mosavari, Nasiri S Khajeh, M Emam, M Ahouran, S Hashemi, Mohammad M Taheri, D Jahanpeyma, S Nikkhah

**Affiliations:** 1 Razi Vaccine and Serum Research Institute; 2 Pasteur Private Veterinary Laboratory; 3 Iran Veterinary Organization

**Keywords:** Glanders, *Burkholderia mallei*, Tiger

## Abstract

**Background and Objectives:**

In December 2010 four, lions and one tiger died at the Tehran zoo. Out of all samples, *Burkholderia mallei* (causative agent of Glanders) was isolated just from ulcer sample of the tiger which was imported to Iran from Russia.

**Materials and Methods:**

One nasal swab from a tiger and fifteen blood samples with anticoagulant belonging to one tiger and fourteen lions (four dead lions and eleven live lions) were collected and were inoculated directly onto the selective media. The isolate was identified by morphological and biochemical and API BBL tests and PCR using specific primers (Bma- IS407-flip). The standard (Razi Type Culture Collection RTCC: 2375) and tiger isolates were inoculated into 2 guinea pigs. All residue solipeds and carnivores were checked by Malleination test and Complement Fixation (CF) Test respectively.

**Results:**

One isolate of *B. mallei* was isolated from tiger's nasal swab. Both of *B.mallei* strains were recovered from inoculated animals. All of solipeds were negative by malleination test, however, 11 lions including 4 dead and 7 live lions out of 14 lions were positive in CF test for Glanders and all were put down by the authorities.

**Conclusion:**

Active surveillance of Glanders is essential for solipeds, especially it's more important while being used to feed valuable carnivores like lions and tigers. Therefore, a reliable test like malleination must be carried out twice (first before transferring and one month after quarantine). Both test results should be negative for use for feeding.

## INTRODUCTION

Glanders is a bacterial contagious zoonotic infection caused by *Burkholderia mallei*. The disease is primarily an infectious disease of the solipeds (horse, mule or donkey), carnivorous (lions, tigers and etc), goats, dogs, rabbits and camels. In humans, glanders usually is acquired through direct skin or mucous membrane contact with infected animal tissues (1).

The disease is occupational and laboratory workers, veterinarian, physician and zoo workers are at high risk. Cases in humans or animals occur sporadically throughout the world. The mortality of untreated infections is high. Although glanders has been eradicated from many countries, the disease is still recognized in the Middle East, Northern Africa, Southeast Asia and Northern Australia (1).

Infections are contracted mainly from highly infectious nasal discharges that contaminate the surroundings, especially harnesses, feeding troughs, and old-fashioned water troughs. Carnivores are usually infected by eating the meat of glanderous horses. A number of serious outbreaks occurred in zoological parks and circuses as a result of feeding horse meat to members of the cat family and last report of the disease belongs to Turkey at the Istanbul zoo. Natural infection may take place by ingestion into the digestive tract, inoculation through the skin, or inhalation into the respiratory tract (2).

*B. mallei* is considered as a potential biological warfare or bioterrorism agent and has been included in the B list of the bioterrorism agents of the Centers for Disease Control and Prevention (3, 4). Glanders was involved in the first modern use of microbes as weapons when German agents targeted horses in the United States, Romania, Spain, Norway, and Argentina between 1915 and 1918. Glanders was reported to have been used during both the first and second world wars. During the First World War, it was used to infect large numbers of Russian horses and mules on the Eastern Front, affecting troop movements. During the Second World War, the Japanese deliberately infected animals and humans at the Pinfang Institute in China (3).

Iran had a national campaign of test and slaughter of infected solipeds by *B. mallei* since 1961 by malleination test. Mallein is produced at the Razi Vaccine and Serum Research Institute for a few years (5). This study was conducted for isolation and identification of the agent which produced disease among lions and tiger at the Tehran zoo.

## MATERIALS AND METHODS

### Case history, isolation and cultural condition

Four African lions and one Siberian tiger passed away at the Tehran zoo in December 2010 with common clinical signs of glanders in carnivores such as depression, anorexia, vomiting, skin ulcers, dyspnea and respiratory disorders.

Pursuant to the mentioned mortality, one nasal swab from one tiger submitted to private Pasteur laboratory on 27^th^ Dec 2010, Tehran, Iran. One bacterial isolate from tiger and fifteen blood samples with anticoagulant belonging to one tiger and fourteen lions from Tehran national zoo park on 12^th^ Jan 2011 submitted to Razi Vaccine & Serum Research Institute, Karaj, Iran.

The samples were inoculated directly onto the 3% glycerol Triptic soy agar (TSA), Triptic soy broth (TSB) and blood-agar (Difco & BBL, NJ USA) and incubated at 37°C in aerobic condition for 48h. The colonies (small, round, convex, translucent, and yellowish) suspected to be *B. mallei* were identified by gram staining, oxidase test, motility tests, TSI, gelatin liquefaction, fermentation of carbohydrates, nitrate reduction and growth at 42°C (6, 7). Identification was confirmed by biochemical profiles on API BBL tests (Difco & BBL, NJ USA). The isolates were stored at −70°C in tryptic soy broth (Difco & BBL, NJ USA) containing 20% glycerol.

### DNA purification and PCR

**Fig. 1:**
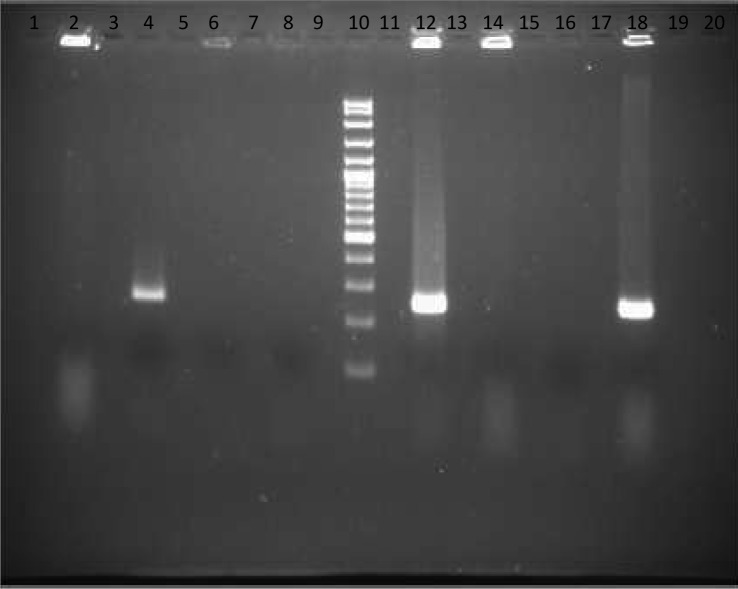
Lanes 1, 3, 5, 7, 9, 11, 13, 15, 17, 19 and 20 are empty, Lane 2: *E. coli*, Lane 4: DNA of Tiger isolates submitted from Pasteure laboratory, Lane 6: *Salmonella enteritidis*, Lane 8: Negative control, Lane 10: 100 bp Size marker, Lane 12: Positive control (Standard strain from Razi Institute), Lane 14: *Pseudomonas aeruginosa*, Lane 16: Negative control, Lane 18: Tiger isolates.

**Fig. 2:**
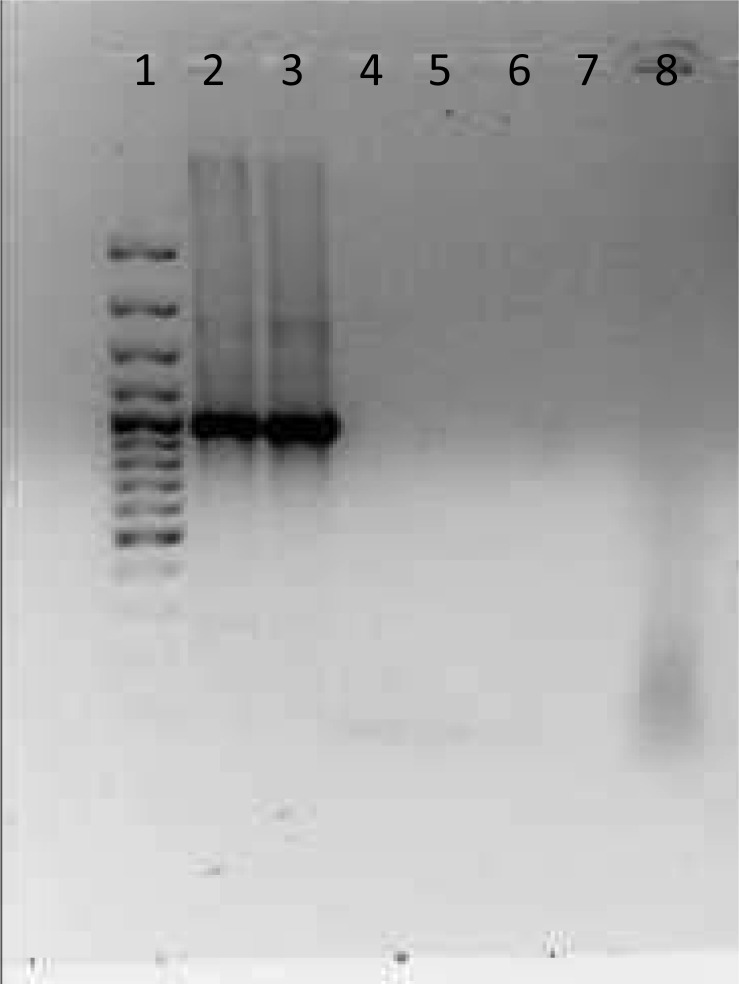
Lane 1: 100 bp Size Marker, Lane 2: Tiger Isolates, Lane 3: Positive control (Standard strain from Razi Institute), Lane 4: *E. coli*, Lane 5:Empty, Lane 6: Negative control, Lane 7: *Salmonella enteritidis*, Lane 8: *Pseudomonas aeruginosa*.

Genomic DNA was extracted by using isoAmyl alchol-chloroform method. Primers Bma-IS407-flip-f (5’-TCA-GGT-TTG-TAT-GTC-GCT-CGG-3’) and Bma- IS407-flip-r (5’-CTA-GGT-GAA-GCT-CTG-CGC-GAG-3’) were used to amplify a 989 bp fragment and another PCR which designed by Pasteur laboratory performed with forward primer (5’-GCG-TTA-AAC-GCC-GTA-CTT-T-< C > -3’) and reverse primer (5’-TTC-GAT-CGA-TTC-CTG-CTA-T-< C > -3’) to amplify a 251 bp fragment (Figs. 1 & 2) (1). Amplification reactions were performed in a 50 µl final volume with ready-to-use mastermix and 15 pmol of each primer. Approximately 80 to 100 ng of DNA template was used in each amplification. The PCR was performed in an Ependorf PCR system with an initial denaturation step of 5 min at 94°C followed by 35 amplification cycles of 30 s at 94°C, 30 s at 65°C and 60 s at 72°C. The samples were then incubated at 72°C for another 7 min and cooled to 4°C.

The amplification products were checked by agarose gel electrophoresis in a 1% agarose gel (Difco & BBL, NJ USA) on a horizontal electrophoresis apparatus (Bio-Rad, TX, USA) at 50 V and 150 mA. Gels were stained with ethidium bromide as described by Sambrook et al (8) and documented digitally with GEL Doc (Bio-Rad, TX, USA). Double-distilled, sterile water instead of template DNA was used as the negative control to exclude amplicon contamination. Positive control containing DNA of *B. mallei* (RTCC 2375) which used for production of Razi mallein test (as standard strain) was included in each run. The sensitivity of the PCR was evaluated by serial dilution of the bacterium and the specificity was analyzed by different genus bacteria such as *Pseudomonas aeruginosa, Salmonella enteritidis* and *E.coli*.

### Inoculums preparation

The tiger isolate and standard strain were cultivated on 3% glycerol TSA medium (Difco & BBL, NJ USA) at 35–37°C. The confluent (48h) growth cultures of two *B. mallei* were suspended in PBS salt solution to achieve a challenge dose of 10^7^ the colony-forming units per milliliter (CFU/ ml) and the optical density were used to determine the CFU/ml. The inoculums were administered IP in two guinea-pigs using a 25-gauge needle.

### Animal inoculation

Two male guinea pigs, weighing between 350 to 400 g, were injected IP with 1 ml of each inoculums. One age- and sex-matched control was injected with 1 ml of PBS salt solution alone. All animals were observed at least twice daily until orchitis (the Strauss reaction) were seen. Each guinea pig located at separate cage in one isolator.

## RESULTS

### Bacterial isolation and biochemical characterization

*B. mallei* was isolated from one tiger's nasal swab. A total of fourteen lions were negative at culture. However the lions were treated by antibiotics. The microscopic and macroscopic morphology were similar to *B. mallei*. The results of biochemical characterizations are shown in Table 1. The results indicated that the isolate was *B. mallei*. The API BBL test also successfully confirmed the results.

**Table 1:** Identification of *B. mallei* and *B. pseudomallei* (2).

Tests	Oxidase	Growth at 42°C	Nitrate Reduction	Gelatin Liquified	Motility	Oxidizes Glucose	Oxidizes Lactose	Oxidizes Mannitol	Arginine dihydrolase	Citrate	TSI	Indol
*B. pseudomallei*	+	+	+	v	+	+	=	=	+	+	Alk/NC1 Gas-, H2S -	-
*B. mallei*	v	=	+	=	-	=	=	=	+	v	Alk/NC1 Gas-, H2S -	-

V = variable; +, > 90% of strain are positive; =, > 90% of strain are negative, NC1 = No Change.

### PCR

PCR amplified 251 and 989 bp fragment for the tiger isolate. Similar fragments were observed for *B. mallei* used for production of the Razi mallein test (standard strain as control positive) (Figs. 1 & 2).

### Animals experiments

**Fig. 3:**
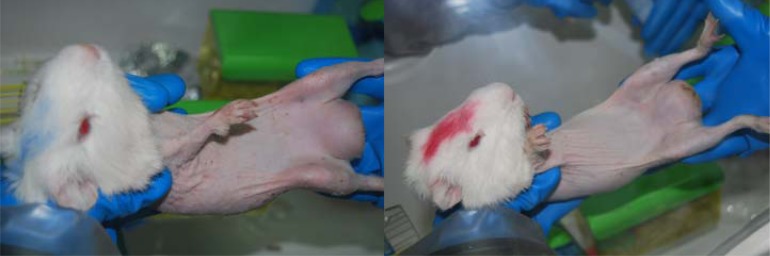
Inoculated guinea pigs by *B. mallei* tiger (Red Mark-Right) and standard (Blue Mark-Left) isolates.

Two infected guinea-pigs and a negative control were euthanized with chloroform three days after inoculation and notification of strauss reaction (Fig. 3). Subsequently, *Burkholderia mallei* was isolated and identified from different tissue of the experimentally infected guinea pigs. Necropsy procedures were conducted as previously described (1, 7). Specimens from testis, spleen, liver, blood and lung were inoculated onto the 3% glycerol TSA and TSB (Difco & BBL, NJ USA) tubes at 35–37°C and PCR test also was performed on the isolated bacteria and the same fragment was observed.

## DISCUSSION

Glanders is a highly contagious and fatal disease. Infected animals can become long-term carriers of *B. mallei* and it can be transmitted to other animals (and people) through close direct contact or contact with oral and nasal secretions and discharge from skin ulcers. It can also be transmitted by eating tissues from infected animals. Prompt euthanasia of affected animals is therefore often the primary means of controlling outbreaks. Glanders can affect species other than equids, including people and cats; however there is very little information available about glanders in any felids including lions and tigers. It might be possible for the disease to spread from the zoo animals to feral cats and then to the zoo cats or people. The zoo cats may also be infected by a glanders-positive equids including donkey or mule may. It is also unclear what tests were used to confirm that the big cats were infected with glanders, and it is unknown if other animals at the zoo have been tested. Since this is typically a disease of equids, these animals should certainly be checked first (1–3).

The big question is where did the glanders come from in the first place? It seems unlikely that the tigers brought it from Russia, when the disease is actually endemic in Iran. Is there a carrier animal in the zoo? Were the animals infected by eating contaminated meat? Was it brought in by feral cats?

One *B. mallei* isolate was isolated only from one tiger and the lions were culture negative. Because of treatment of lions by antibiotic, we couldn't isolate the bacterium but the CF test was positive. These results suggested that to resolve this controversy, the isolation should be performed before initiation of antibiotic therapy.

Based on reports and evidence, soliped meats were used for feeding all carnivores without any screening tests for glanders (2). After the outbreak, all of the residues of zoo solipeds were checked for glanders and were negative. This observation indicates that the infection probably originated from prepared solipeds for feeding of carnivores including tigers and lions.

There was not any active surveillance for screening of feeding of carnivores with soilpeds in Iran's zoo. Because *B. mallei* is a highly pathogenic agent, the monitoring of the soilpeds is necessary by a reliable test like maleination test done twice. The first test should be done when they are prepared for zoo entry and the second test is after animals are quarantined at least for 30 days. When both test results are negative, they can be used for feeding. It is strictly recommended that “meat not be used if animals is suspected or is positive for feeding carnivores”. This monitoring is very important for other Iranian zoos in different areas.
